# All-Trans-Retinoic Acid Modulates ICAM-1 N-Glycan Composition by Influencing GnT-III Levels and Inhibits Cell Adhesion and Trans-Endothelial Migration

**DOI:** 10.1371/journal.pone.0052975

**Published:** 2012-12-26

**Authors:** Changguo Chen, Dekun Diao, Liang Guo, Ming Shi, Jie Gao, Meiru Hu, Ming Yu, Lu Qian, Ning Guo

**Affiliations:** 1 Department of Pathophysiology, Institute of Basic Medical Sciences, Beijing, P.R. China; 2 Department of Clinical Laboratory, the Navy General Hospital, No. 6 Fucheng Road, Beijing, P.R. China; 3 Laboratory of Cellular and Molecular Immunology, Medical School of Henan University, Kaifeng, P.R. China; 4 Institute of Basic Medicine, Shandong Academy of Medical Science, Jinan, P.R. China; Southern Illinois University School of Medicine, United States of America

## Abstract

Changes in the expression of glycosyltransferases directly influence the oligosaccharide structures and conformations of cell surface glycoproteins and consequently cellular phenotype transitions and biological behaviors. In the present study, we show that all-trans-retinoic acid (ATRA) modulates the *N*-glycan composition of intercellular adhesion molecule-1 (ICAM-1) by manipulating the expression of two *N*-acetylglucosaminyltransferases, GnT-III and GnT-V, via the ERK signaling pathway. Exposure of various cells to ATRA caused a remarkable gel mobility down-shift of ICAM-1. Treatment with PNGase F confirmed that the reduction of the ICAM-1 molecular mass is attributed to the decreased complexity of *N*-glycans. We noticed that the expression of the mRNA encoding GnT-III, which stops branching, was significantly enhanced following ATRA exposure. In contrast, the level of the mRNA encoding GnT-V, which promotes branching, was reduced following ATRA exposure. Silencing of GnT-III prevented the molecular mass shift of ICAM-1. Moreover, ATRA induction greatly inhibited the adhesion of SW480 and U937 cells to the HUVEC monolayer, whereas knock-down of GnT-III expression effectively restored cell adhesion function. Treatment with ATRA also dramatically reduced the trans-endothelial migration of U937 cells. These data indicate that the alteration of ICAM-1 *N*-glycan composition by ATRA-induced GnT-III activities hindered cell adhesion and cell migration functions simultaneously, pinpointing a unique regulatory role of specific glycosyltransferases in the biological behaviors of tumor cells and a novel function of ATRA in the modulation of ICAM-1 *N*-glycan composition.

## Introduction

Intercellular adhesion molecule-1 (ICAM-1) belongs to the immunoglobulin superfamily of adhesion molecules [1]. ICAM-1 functions to promote intercellular contact/adhesion and to induce trans-endothelial migration of leukocytes. ICAM-1 also acts as a co-stimulatory molecule and signal transducer to trigger intracellular signals, ultimately leading to the activation of lymphocytes, secretion of cytokines and induction of proinflammatory cascades [2], [3]. ICAM-1 is a heavily *N*-glycosylated transmembrane protein with a molecular weight of 80–114 kDa depending on its level of glycosylation [1], [4]. The various functions of ICAM-1 appear to be differentially regulated by *N*-linked glycosylation [5]. Alterations in the oligosaccharide structure (glycan) of ICAM-1 may significantly influence cell proliferation, differentiation, adhesion, migration, tumor invasion and metastases [6], [7], [8], [9].

*N*-glycan processing or remodeling reactions are catalyzed by several glycosyltransferases, which generate highly diverse *N*-linked glycans (*N*-glycans) [10], [11]. Among the glycosyltransferases, two major glycosyltransferases are Golgi *N*-acetylglucosaminyltransferase III (GnT-III) and V (GnT-V). GnT-III catalyzes the formation of a bisecting β1,4-GlcNAc structure, whereas GnT-V promotes the synthesis of a β1,6-branching GlcNAc structure [12], [13]. GnT-III is considered to be a key glycosyltransferase in the *N*-glycan biosynthetic pathway because the introduction of the bisecting GlcNAc residue suppresses further processing and elongation of the *N*-glycans catalyzed by GnT-V. GnT-V is unable to utilize the bisected oligosaccharide as a substrate [14], [15].

Retinoids, a family of retinol metabolites and synthetic derivatives, exert diverse functions. These functions include regulating growth and differentiation in different cell types [16], [17], [18], inhibiting malignant cell proliferation and modulating the expression of vascular cell adhesion molecule-1 (VCAM-1) and ICAM-1 [19], [20], [21]. Retinoids have long been applied to cancer chemoprevention [8], [22]. Retinoic acid (RA) and its analogues have also been used in topical and systemic treatment of inflammatory skin diseases because the regulated expression of cell adhesion molecules is critical for leukocyte recognition and transmigration through the endothelium during inflammatory responses.

In the present study, we found that all-trans-retinoic acid (ATRA) modulates the *N*-glycan composition of ICAM-1 by up-regulating GnT-III and down-regulating GnT-V via activation of the ERK signaling pathway. The changes mediated by ATRA inhibit cell adhesion and trans-endothelial migration, pinpointing a unique regulatory role of specific glycosyltransferases in the biological behaviors of tumor cells and highlighting the novel ATRA function of modulating the *N*-glycan composition of ICAM-1.

## Results

### ATRA induces the expression of ICAM-1 and modifies its *N*-glycan structure

**Figure 1:**
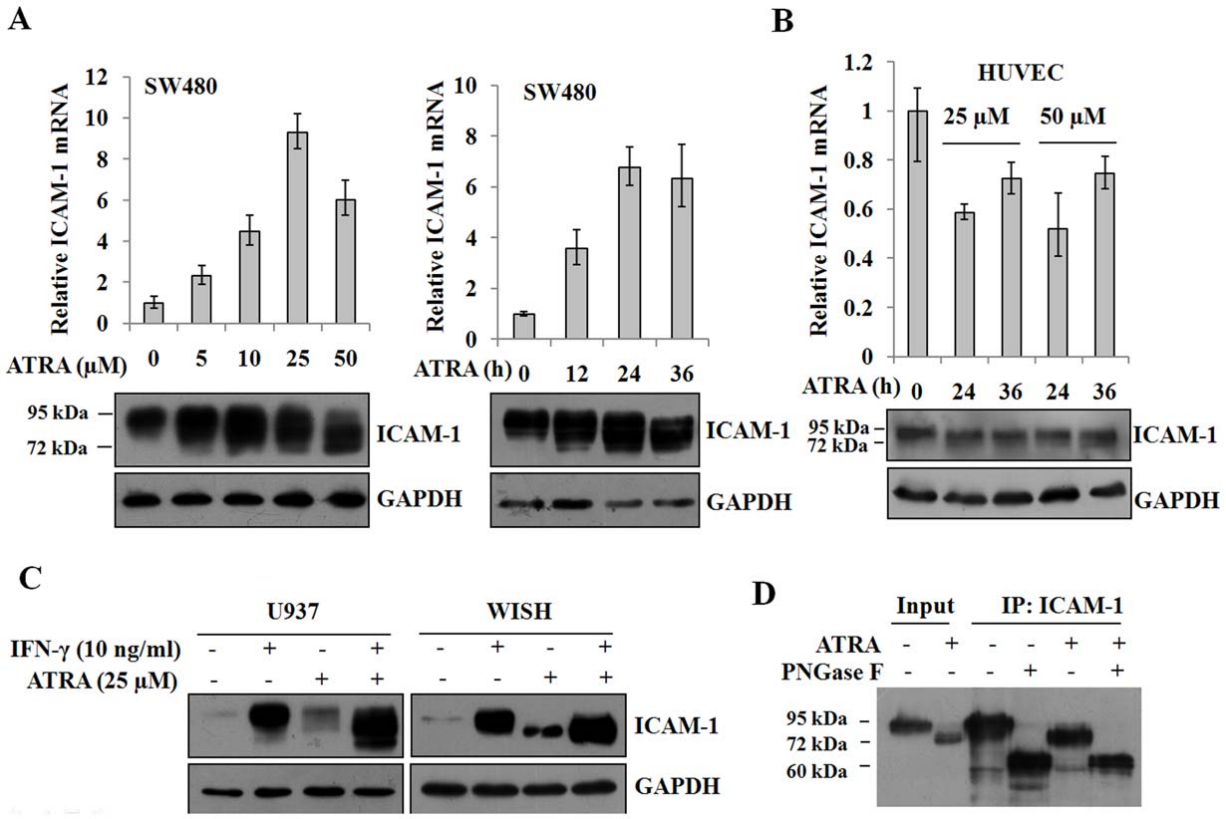
ATRA induces the expression and *N*-glycan modification of ICAM-1. A, SW480 cells were treated with 0, 5, 10, 25 and 50 µM ATRA for 36 h or with 25 µM ATRA for 0, 12, 24 and 36 h. The expression of ICAM-1 was analyzed by real-time RT-PCR (n = 3) and Western blot. Equal loading was verified by detection of GAPDH. B, HUVEC cells were treated with 25 or 50 µM ATRA for 0, 24 and 36 h. The expression of ICAM-1 was analyzed by real-time RT-PCR (n = 3) and Western blot. C, U937 and WISH cells were treated with either 10 ng/ml IFN-γ or 25 µM ATRA or both. The expression of ICAM-1 was analyzed by Western blot. D, WISH cells were treated with 25 µM ATRA. The cell lysates were immunoprecipitated by the antibody against ICAM-1 and then treated with PNGase F. The digested samples were blotted and detected by Western blot.

ICAM-1 is usually expressed at low levels on vascular endothelium, lymphocytes and macrophages, but its expression can be induced in response to inflammatory stimuli [23]. Several studies reported that ICAM-1 expression was upregulated by RA [6], [24]. When SW480 cells were treated with ATRA, the mRNA and protein expression levels of ICAM-1 were strongly enhanced (Figure 1A). Interestingly, ICAM-1 exhibited a diffuse pattern in a gel mobility assay, with bands extending from a molecular weight of ∼95 kDa to ∼72 kDa in a dose- and time-dependent manner. A similar change was also observed in HUVEC cells, though the expression of ICAM-1 in HUVEC cells was not markedly induced by ATRA (Figure 1B).

Previous reports have demonstrated that the expression of the *ICAM-1* gene is regulated by RA [24]. However, the molecular weight shift of ICAM-1 following ATRA induction has not been reported. To verify whether the ICAM-1 gel mobility shift was specifically correlated with ATRA induction, we treated U937 and WISH cells, which express low levels of ICAM-1, with IFN-γ. IFN-γ induces the expression of ICAM-1 [23], [25]. IFN-γ significantly induced the expression of ICAM-1 in both cells, but induction did not result in an ICAM-1 gel mobility down-shift (Figure 1C). Interestingly, ATRA treatment not only markedly promoted the expression of ICAM-1, but it also caused a reduction in the molecular weight of ICAM-1. Simultaneous stimulation with ATRA and IFN-γ led to even an greater expression of ICAM-1 and a pronounced molecular mass shift in U937 and WISH cells (Figure 1C), indicating that the effect of ATRA is not cell line-specific.

Mature ICAM-1 is a highly glycosylated protein in cells. Our data suggested that the ICAM-1 gel mobility shift could be due to changes in its glycosylation status. To test this hypothesis, lysates from WISH cells that were treated with ATRA were immunoprecipitated and then treated with PNGase F, an enzyme that removes virtually all types of *N*-glycans from glycoproteins. As shown in Figure 1D, the PNGase F enzymatic treatment completely released the oligosaccharide chains and the deglycosylated products from both ATRA-treated and control cells. In these cells, the molecular weight of ICAM-1 shifted to the same position of ∼60 kDa, suggesting that the number and/or size of *N*-glycans linked to ICAM-1 decreased following ATRA induction.

### Endoplasmic reticulum (ER) stress is not the main cause for alteration of ICAM-1 *N*-glycan modification

**Figure 2:**
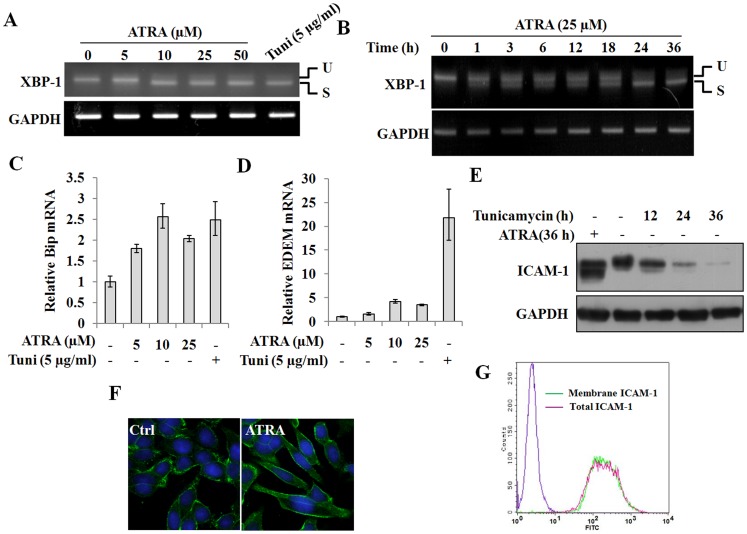
ER stress is not the main cause for alteration of ICAM-1 *N*-glycan composition. A, Total RNA was isolated from SW480 cells that treated with 0, 5, 10, 25 and 50 µM ATRA or 5 µg/ml Tunicamycin. The expression of XBP-1 at the mRNA level was assessed by RT-PCR. B, SW480 cells were treated with 25 µM ATRA for 0, 1, 3, 6, 12, 18, 24 and 36 h. The expression of XBP-1 at the mRNA level was assessed by RT-PCR. C and D, SW480 cells were treated with 0, 5, 10 and 25 µM ATRA or with 5 µg/ml tunicamycin for 36 h. The expression of BiP (C) and EDEM (D) at the mRNA levels was evaluated by real-time RT-PCR (n = 3). E, SW480 cells were treated with 25 µM ATRA for 36 h or with 5 µg/ml tunicamycin for 12, 24 and 36 h. The expression of ICAM-1 was analyzed by Western blot. F, SW480 cells were grown on coverslips for 12 h and then incubated with 25 µM ATRA for 36 h. Then the cells were labeled with the anti-ICAM-1 antibody and FITC-conjugated goat anti-mouse IgG and examined under a confocal microscope. G, SW480 cells were treated with 25 µM ATRA for 48 h. The expression of ICAM-1 on the cell membranes was analyzed by flow cytometry. For the analysis of total ICAM-1 expression in the cells, the cells were fixed, permealized and stained. The stained cells were analyzed by flow cytometry.

*N*-glycosylated proteins represent the majority of polypeptides expressed in the ER [26]. A previous study reported that ATRA intensified ER stress in human hepatocarcinoma cells that were transfected with a GnT-V antisense oligonucleotide [27]. To determine whether the ATRA-mediated modification of ICAM-1 *N*-glycan structures is associated with ER stress, we treated SW480 cells with 25 µM ATRA and analyzed the transcription of X-box-binding protein (XBP-1), a transcription factor that can be spliced by the IRE1 kinase/endoribonucleas upon ER stress. Splicing of XBP-1 results in the removal of a 26-nucleotide intron [28], [29]. Tunicamycin prevents the synthesis of the glycosylated lipid precursor of *N*-glycans and was used as a control. We observed the spliced mRNA band (88 bp), which appeared in a dose- and time-dependent manner (Figures 2A and 2B). This result indicates that XBP-1 was spliced following ATRA treatment. In agreement with a previous study, ATRA induced BiP expression at the mRNA level (Figure 2C). BiP is a marker for the ER stress response [30], and its induction confirms that ATRA induction triggers ER stress. Unlike tunicamycin, which triggers transcriptional upregulation of a stress-regulated protein termed ER degradation enhancing α-mannosidase like protein (EDEM) [29], [31], the effect of ATRA on EDEM expression was only marginal (Figure 2D). Treatment with tunicamycin not only altered the size of the ICAM-1 protein, but it also induced ER-associated degradation (ERAD) of ICAM-1 in a time-dependent manner (Figure 2E). Conversely, ATRA treatment resulted in an accumulation of ICAM-1 (Figure 2E), suggesting that the mechanisms of ATRA-induced modification of the ICAM-1 *N*-glycosylation status are different from the mechanisms of tunicamycin-mediated *N*-glycosylation inhibition. The ER tightly controls protein products, and only proteins in their native state can be transported to the Golgi prior to arriving at their appropriate cellular location [32]. We conducted fluorescent confocal microscopy and flow cytometry experiments to determine whether ATRA induction affects the intracellular transport of ICAM-1. As shown in Figures 2F and 2G, ICAM-1 was predominantly located at the cytoplasmic membrane in both ATRA-treated and control cells, demonstrating that ICAM-1 molecules were transported and properly localized within the cell. These data demonstrate that ER stress is not the main cause of altered ICAM-1 *N*-glycan composition.

### ATRA-induced GnT-III activity is involved in changes of ICAM-1 *N*-glycan composition

**Figure 3:**
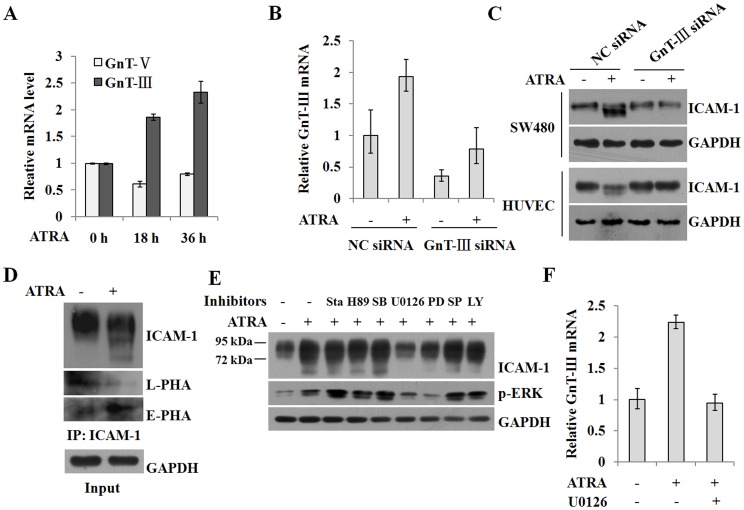
ATRA-induced GnT-III expression is involved in the modulation of ICAM-1 *N*-glycan composition. A, SW480 cells were treated with 25 µM ATRA for 0, 18 and 36 h. The expression of GnT-III and GnT-V at the mRNA levels was detected by real-time RT-PCR (n = 3). B and C, SW480 cells that were transiently transfected with 50 nM of the siRNA specifically targeting GnT-III were treated with 25 µM ATRA. The efficiency of transfection was analyzed by real-time RT-PCR (n = 3, B) and the expression of ICAM-1 by Western blot (C). D, SW480 cells were treated with 25 µM ATRA. Then immunoprecipitation by the antibody against ICAM-1 (1.5 µg per 500 µg of total protein) was performed. The immunoprecipitated products were subjected to 10% SDS-PAGE, transferred to a nitrocellulose membrane and consecutively incubated with biotinylated L-PHA or E-PHA lectin, streptavidin-labled rabbit IgG and HRP-labeled goat anti-rabbit IgG. Bound HRP on the membranes was detected by ECL. E, SW480 cells were pretreated with 0.7 nM stauroporine, 10 µM H-89, 10 µM SB203580, 10 µM U0126, 50 µM PD98059, 20 µM SP600125 or 50 µM LY294002 for 2 h and then exposed to 25 µM ATRA for 36 h. The expression of ICAM-1 and phosphorylation of ERK were analyzed by Western blot. F, SW480 cells were pretreated with 10 µM U0126 and then with 25 µM ATRA. The expression of GnT-III at the mRNA level was analyzed by real-time RT-PCR (n = 3).

Glycosyltransferases GnT-III and GnT-V participate in the processing of *N*-glycans during the synthesis of glycoproteins. To determine which of these enzymes contributes to the ATRA-induced modification of ICAM-1 *N*-glycan composition, we analyzed the levels of the mRNAs encoding these enzymes. Using conventional culture conditions, the expression of these two enzymes was nearly identical (Figure 3A). Interestingly, ATRA induction resulted in a marked increase of GnT-III levels in addition to a decrease in GnT-V levels, in accordance with a reduction in ICAM-1 molecular weight. To investigate the role of GnT-III in this process, we used a siRNA targeting GnT-III. Figure 3B demonstrates that the expression of GnT-III was successfully silenced by the specific siRNA and that ATRA-induced upregulation of GnT-III expression was also abrogated. The molecular weight of ICAM-1 was also restored (Figure 3C), confirming that the reduced number and size of *N*-glycans accounts for the smaller molecular weight of ICAM-1.

To identify the *N*-glycan structures of ICAM-1, we utilized lectin L-phytohemagglutinin (L-PHA), which specifically recognizes β1,6 branched structures (a product of GnT-V), and E-PHA lectin, which recognizes bisecting GlcNAc structures (a product of GnT-III). ATRA induction resulted in an increase in the GnT-III product, as revealed by a marked increase in E-PHA reactivity and a decrease in L-PHA reactivity (Figure 3D).

To determine the signaling pathways involved in ATRA-induced alterations of ICAM-1, we utilized various signaling pathway inhibitors. Figure 3E shows that inhibition of ERK signaling by U0126 and PD98059 prominently blocked the gel mobility shift of ICAM-1, and ATRA-induced ICAM-1 expression was also impaired following ERK inhibition. In addition, chemical inhibition of ERK signaling significantly repressed the transcription of the gene encoding GnT-III (Figure 3F). The data suggest that ATRA induced the alteration of ICAM-1 *N*-glycan composition by regulating the activities of GnT-III via activation of the ERK signaling pathway.

### Inhibition of ICAM-1 *N*-glycan elongation or processing by ATRA suppresses cell adhesion and trans-endothelial migration

**Figure 4:**
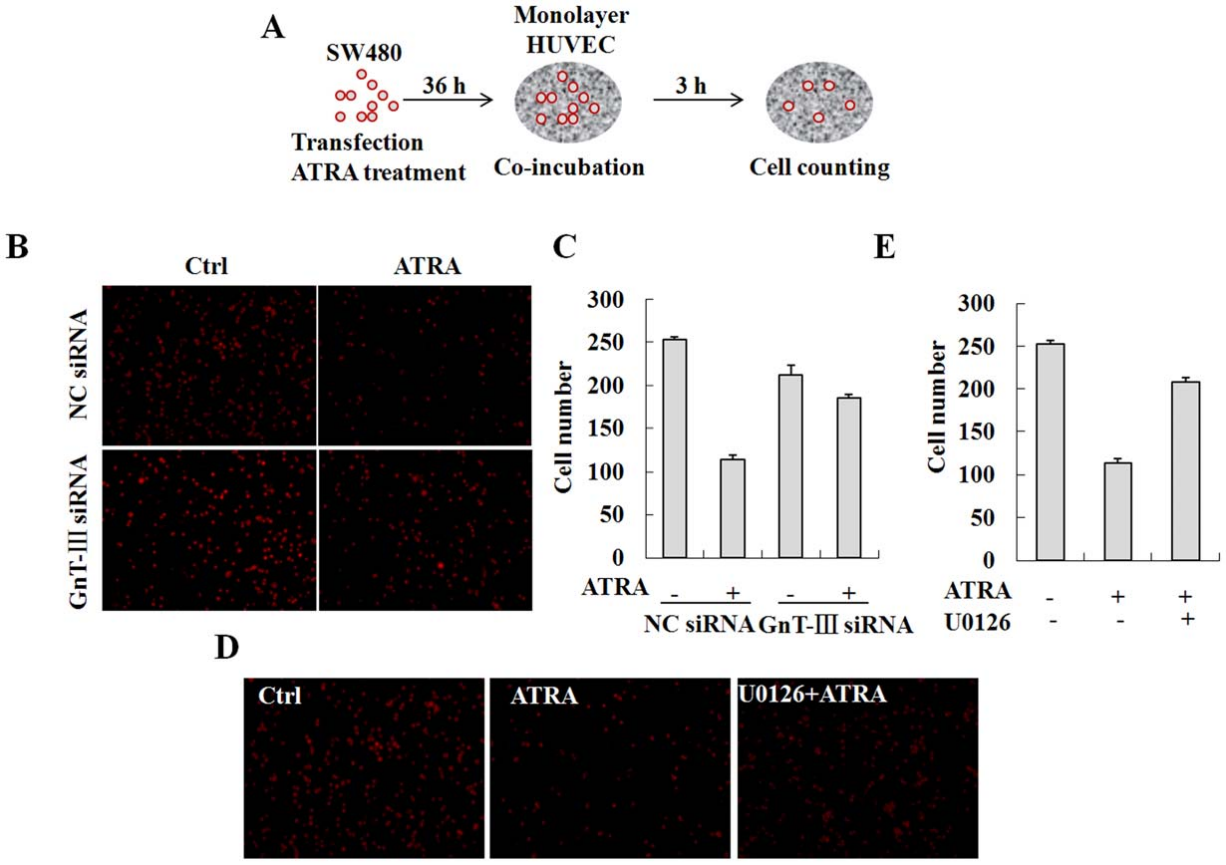
Inhibition of ICAM-1 *N*-glycan elongation or processing by ATRA suppresses cell adhesion. A, Schematic presentation of the protocol used to determine the effect of ATRA on cell adhesion. Briefly, SW480 cells were transfected with the GnT-III specific siRNA, treated with ATRA and then co-incubated with the HUVEC monolayer. The cell adhesion was assessed by counting the cells attached to the HUVEC monolayer. B, The cells attached to the HUVEC monolayer were observed under a confocal microscope. C, The adherent cells were analyzed by cell counting. D, SW480 cells were pretreated with 10 µM U0126 and then with 25 µM ATRA. The cells attached to the HUVEC monolayer were observed under a confocal microscope. E, The adherent cells were analyzed by cell counting.

The structure of *N*-glycans that are linked to adhesion molecules may affect intercellular adhesion. Previous reports have shown that reduced branching of *N*-glycans promotes cell adhesion in epithelia [13], [33]. Additionally, decreased expression of *N*-linked β1,6-branched glycans on N-cadherin enhances N-cadherin-mediated cell-cell adhesion by depletion of GnT-V in HT1080 fibrosarcoma cells [34]. To determine the functional relevance of the modified ICAM-1 *N*-glycan composition, SW480 cells were transfected with the GnT-III specific siRNA, treated with ATRA and then co-incubated with a HUVEC monolayer. Cell adhesion was assessed in these ATRA-treated and GnT-III depleted cells by counting the cells attached to the HUVEC monolayer (Figure 4A). In contrast to previous studies, adhesion of the control SW480 cells to the HUVEC monolayer was significantly reduced in the presence of ATRA, which inhibited *N*-glycan branching (Figures 4B and 4C). Moreover, knock-down of GnT-III expression in SW480 cells led to a reversal of adhesion potential in these cells. In addition, inhibition of the ERK signaling pathway by U0126 reversed ATRA-induced repression of cell adhesion (Figure 4D and 4E). These results indicate that inhibition of ICAM-1 *N*-glycan branching reduced cell adhesion and that activation of ERK signaling is required in this process.

**Figure 5:**
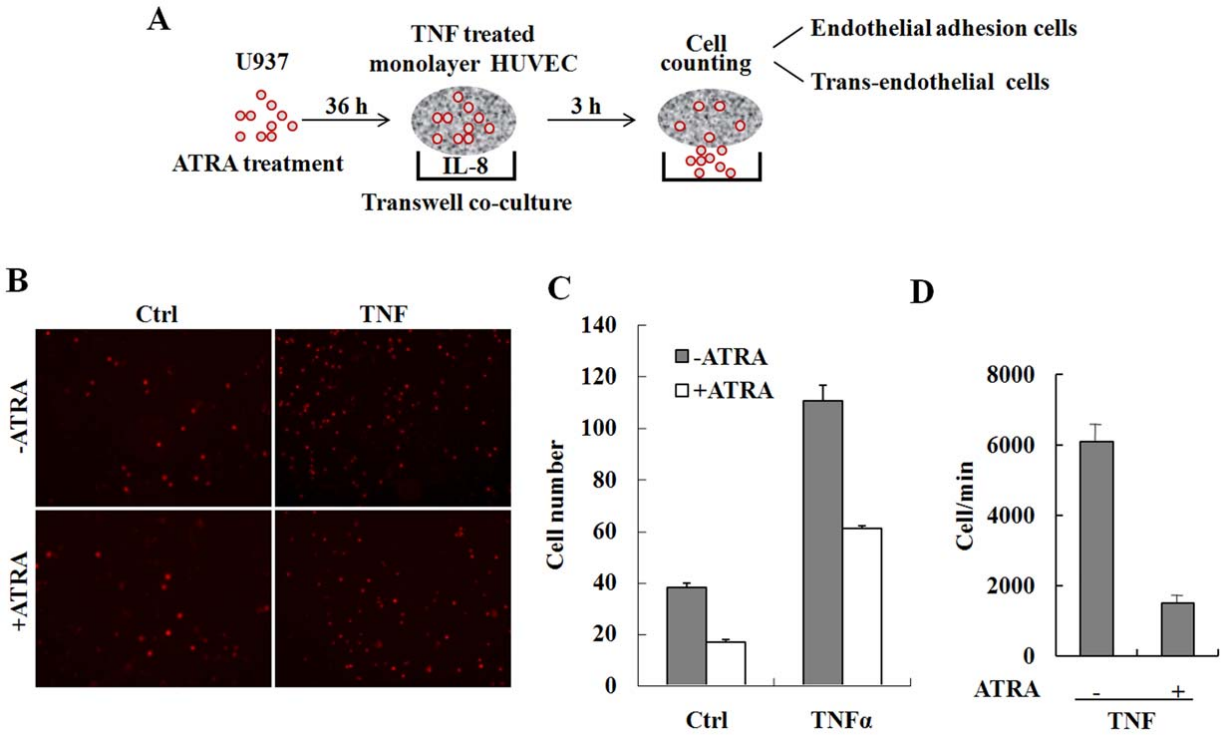
Inhibition of ICAM-1 *N*-glycan elongation or processing by ATRA suppresses trans-endothelial migration of U937 cells. A, Schematic presentation of the protocol used to determine the effect of ATRA on trans-endothelial migration of U937 cells. Briefly, U937 cells were treated with ATRA for 36 h and then co-incubated with the confluent monolayer of HUVEC cells, which were treated with 10 ng/ml TNFα and cultured on matrigel-coated transwell filters. 50 ng/ml IL-8 were added to the lower chamber. B to D, U937 cells attached to the HUVEC monolayer and trans-migrated through the matrix-membrane unit were estimated by confocal microscopy (B), cell counting (C) and flow cytometry (D).

To further investigate the effect of ATRA on cell adhesion and trans-endothelial migration, U937 cells were treated with ATRA for 36 h and then co-incubated with a confluent monolayer of HUVEC cells, which were treated with 10 ng/ml TNFα and cultured on matrigel-coated transwell filters. IL-8 (50 ng/ml), a potent chemoattractant for neutrophil trans-endothelial migration was added to the lower chamber [35]. U937 cells that trans-migrated through the matrix-membrane unit and attached to the HUVEC monolayer were estimated by flow cytometry and cell counting (Figure 5A). The data demonstrate that adhesion of U937 cells was greatly promoted by TNFα stimulation in the absence of ATRA. However, ATRA treatment significantly impaired the positive effect of TNFα (Figures 5B and 5C). Surprisingly, the trans-endothelial migration of U937 cells was also strongly inhibited (Figure 5D). The results show that the alteration of the ICAM-1 *N*-glycan composition reduces cell adhesion and trans-endothelial migration simultaneously.

## Discussion

**Figure 6:**
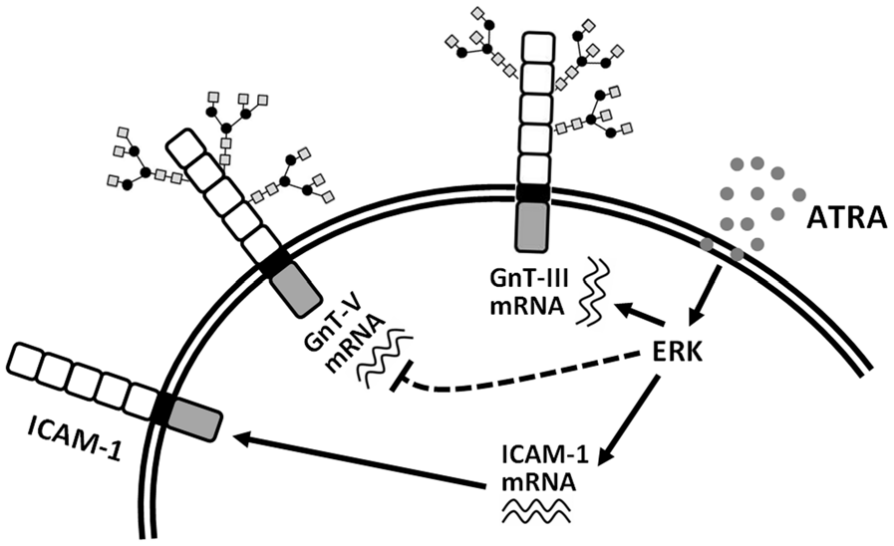
Schematic presentation of the mechanisms by which ATRA modulates the structure of *N*-glycans linked to ICAM-1.

In the present study, we show that ATRA modulates the *N*-glycan structures linked to ICAM-1 by manipulating the expression of GnT-III via the ERK signaling pathway (Figure 6), which consequently suppresses intercellular adhesion and trans-endothelial migration. Exposure of various cells to ATRA caused a remarkable gel mobility down-shift of ICAM-1. Treatment of cells with PNGase F confirmed that the reduction in ICAM-1 molecular mass is attributed to the decreased complexity of *N*-glycans. We also observed increased BiP protein expression and spliced XBP-1 mRNA, though ATRA-induced ER stress was not the major mechanism for these modified ICAM-1 *N*-glycan structures. We noticed that expression of the mRNA encoding GnT-III was significantly enhanced following ATRA induction. In contrast, expression of the mRNA encoding GnT-V was reduced. The expression pattern of these two enzymes reflects the alteration of ICAM-1 *N*-glycan structures. Furthermore, depletion of GnT-III prevented the molecular weight shift of ICAM-1, indicating that GnT-III plays a key role in ATRA-induced ICAM-1 *N*-glycan modification.

Changes in the expression level of glycosyltransferases directly influence the oligosaccharide structures and conformations of cell surface glycoproteins, thus modifying cellular phenotype transitions and biological behaviors [27], [36], [37]. The extracellular domain of ICAM-1 is essential for intercellular adhesion and transendothelial migration of leukocytes [7], [38], [39]. Changes in the *N*-glycan profile of ICAM-1 on the cell membrane are expected to interfere with ICAM-1-mediated cellular functions, such as cell adhesion and migration. When SW480 cells were treated with ATRA, cell adhesion to a HUVEC monolayer was greatly inhibited. Depletion of GnT-III expression with a specific siRNA effectively restored cell adhesion function. Treatment with ATRA also dramatically reduced the trans-endothelial migration of U937 cells. These data indicate that modification of the *N*-glycan structures of ICAM-1 by ATRA-induced GnT-III led to altered cell adhesion and cell migration simultaneously. The precise molecular mechanisms of how ATRA regulates these two processes require further investigation.

Aberrant *N*-glycosylation has been associated with virtually all types of cancer [11], [13], [40]. Increased cell surface complex-type *N* ***-***linked glycans may be associated with phenotypic alterations in transformed cells and invasive potential in tumor cells [41]. Furthermore, GnT-V activity and 1,6 branched *N-*glycan levels are elevated in highly metastatic tumor cell lines [36], [42], [43], [44]. Conversely, GnT-III was considered to be a metastatic suppressor [45]. However, metastasis in a human cervical cancer cell line that overexpresses GnT-III was also reported [37], and elevated GnT-III activity was observed in hepatic neoplasia and several hepatoma cell lines [46]. These data suggest that GnT-III may promote or suppress tumor progression depending on certain conditions. Whether GnT-III performs its functions in a cell-type specific manner is unclear.

The interaction between tumor cells and lymphocytes and endothelial cells via ICAM-1 is complex. On one hand, adherence of tumor cells to endothelium triggers inflammatory cytokine release, which is necessary for inhibiting tumor growth. In contrast, the substances secreted by activated immune cells break down endovascular and endolymphatic barriers, thus permitting trans-endothelial tumor cell migration, which is a crucial step in tumor metastasis. Elevated expression of ICAM-1 has been associated with tumor progression in certain types of cancers [6], [9], [47], [48]. However, in some primary and secondary cancer cells, the ICAM-1 expression is lower than in normal cells [49], [50]. The surface expression of ICAM-1 in breast and cervical cancer cell lines was selectively up-regulated by ATRA [8], [22]. The effect of ATRA on cell adhesion has been contradictory in the literature. Alteration of ICAM-1 expression or modification to *N*-glycan structures could impact cellular functions of these cell adhesion molecules. It is important to understand how the expression and *N*-glycan composition of ICAM-1 are regulated by ATRA in different cell types. Moreover, understanding the underlying mechanisms and resolving the glycan structures produced upon ATRA induction will shed new light onto the functional versatility of ICAM-1 in tumor progression.

## Materials and Methods

### Cell culture and treatments

Human colon cancer cell line SW480, human amnion-derived WISH cells and human myelomonocytic leukemia U937 cells were obtained from the American Type Culture Collection. SW480 cells were cultured in Iscove's modification of Dulbecco's medium (IMDM) (GIBCO) supplemented with 10% fetal bovine serum (FBS). WISH cells were grown in Dulbecco's modified Eagles medium (DMEM) (GIBCO) with 10% FBS. U937 cells were maintained in RPMI 1640 medium (GIBCO) supplemented with 10% FBS. Human umbilical vein endothelial cells (HUVEC) were cultured by a modified method according to Jaffe et al [51]. SW480 cells were treated with 0, 5, 10, 25 and 50 µM ATRA (Sigma) for 36 h or with 25 µM ATRA for 0, 12, 24 and 36 h. HUVEC cells were treated with 25 or 50 µM ATRA for 0, 24 and 36 h. U937, HUVEC and WISH cells were treated with either 10 ng/ml IFN-γ (PeproTech) or 25 µM ATRA or both. To determine signaling pathways involved, cells were pretreated with 0.7 nM PKC inhibitor stauroporine, 10 µM PKA inhibitor H-89, 10 µM p38 inhibitor SB203580, 10 µM MEK1/2 inhibitor U0126, 50 µM MEK1 inhibitor PD98059, 20 µM JNK inhibitor SP600125 or 50 µM PI3K inhibitor LY294002 for 2 h and then exposed to 25 µM ATRA for 36 h. To determine cellular ER stress, cells were incubated with 5 µg/ml Tunicamycin (Sigma) for the indicated time points.

### Western blot

The whole-cell lysates were prepared, separated by 10% SDS-PAGE and transferred to polyvinylidene difluorid membranes (Amersham). After blocking, the membranes were incubated with the mouse monoclonal antibodies against ICAM-1 (1∶500 dilution in blocking buffer, Santa Cruz) or p-ERK (1∶1000 dilution in blocking buffer, CST), followed by washing and incubating with 1∶5000 diluted horseradish peroxidase (HRP)-conjugated secondary antibodies (Dingguo, China). Eaqual loading was verified by detection of glyceraldehyde-3-phosphate dehydrogenase (GAPDH) using the specific mouse monoclonal antibody (1∶1000 dilution in blocking buffer, Kangchen, China). Bands were detected by ECL (Pierce). All experiments were performed by duplicate.

### GnT-III gene silencing

The siRNA (5′-CGTCAACCACGAGTTCGACCT-3′) targeting GnT-III mRNA was purchased from Ribobio (China). Non-silencing siRNA duplexes were used as a negative control. SW480 cells were transiently transfected with 50 nM of siRNAs using the Lipofectamine® RNAiMAX reagent (Invitrogen). The efficiency of transfection was detected by real-time RT-PCR. A parallel sample of siRNA-treated cells was used for Western blot analysis of the gel mobility shift of ICAM-1. Two independent transfection experiments were performed.

### Conventional and real-time RT-PCR

Total RNA was isolated from SW480 cells treated with ATRA or Tunicamycin using Trizol (Invitrogen) according to the manufacturer's instructions and quantified by spectrophotometry. For amplification of XBP-1, cDNA was synthesized from 2 µg of total RNA using random hexamer primers (RevertAidTM First Strand cDNA Synthesis Kit, Fermentas) as recommended by the manufacturer and subsequently amplified by PCR. For detection of ICAM-1, BiP, EDEM, GnT-III and GnT-V, real-time PCR was performed using SYBR green PCR core reagent (Applied Biosystems) on Real-Time PCR Detection System (Stratagene M×300P). The primers used are listed in Table 1. The results were analyzed using the comparative threshold cycle method with GAPDH as an internal control. The experiment was conducted in triplicate.

**Table 1:** The primers used.

Amplification of	Primers	
XBP-1	Forward	5′-CCAAGGGGAATGAAGTGA-3′
	Reverse	5′-GGGAGATGTTCTGGAGGG-3′
ICAM-1	Forward	5′-ACGGTGCTGGTGAGGAGA-3′
	Reverse	5′-GCTGGCAGGACAAAGGTC-3′
EDEM	Forward	5′-AGGTGCTGATAGGAGATG-3′
	Reverse	5′-TGCCTGGTAGAGGAGATA-3′
Bip	Forward	5′-ACGTGGGCACGGTGGTCG-3′
	Reverse	5′-GTGTTCTCGGGGTTGGAG-3′
GnT-III	Forward	5′-CCGCCACAAGGTGCTCTAT-3′
	Reverse	5′-GATCTCGTCCGCATCGTCA-3′
GnT-V	Forward	5′-ACCATCCAGCAGCGAACT-3′
	Reverse	5′-CCGTCCATAGGAGGCAAT-3′
GAPDH	Forward	5′-ACCCAGAAGACTGTGGATGG-3′
	Reverse	5′-CAGTGAGCTTCCCGTTCAG-3′

### Immunoprecipitation and peptide N-glycosidase F (PNGase F) digestion

WISH cells were treated with 25 µM ATRA. After washing with ice-cold phosphate-buffered saline (PBS), the cells were harvested in lysis buffer (50 mM Tris-HCl, pH 7.4, 150 mM NaCl, 1 mM EDTA, 1 mM EGTA, 1% (w/v) NP-40, 10% (w/v) glycerol, 0.5 mM sodium orthovanadate). The cell lysates were incubated with the antibody against ICAM-1 (2 µg per 500 µg of total protein in 1 ml of cell lysate) and then protein A/G PLUS-Agarose (Santa Cruz). The immunoprecipitated samples were treated with PNGase F (New England Biolabs) according to the manufacture's protocols. A parallel experiment was conducted under the same conditions, except that PNGase F was omitted. Following termination of the reaction, the samples were blotted and detected with the anti-ICAM-1 antibody. This experiment was performed by duplicate.

To analyze the ICAM-1 *N*-glycan structures, SW480 cells were treated with 25 µM ATRA and then immunoprecipitation by the anti-ICAM-1 antibody was performed. The precipitated products were subjected to 10% SDS-PAGE and transferred to nitrocellulose membranes. Biotinylation of L-PHA (Sigma) and E-PHA (Sigma) was carried out with N-hydroxysuccinimide biotin (Sigma) according to the previously reported method [52]. After blocking, the membranes were incubated with biotinylated L-PHA or E-PHA (1 µg/ml) for 1 h at room temperature, unbound lectins removed by washing and the membranes subsequently incubated with streptavidin-labled rabbit IgG (1∶200 dilution, Earthox) for 1 h at room temperature, followed by washing. Then the membranes were incubated with HRP-labeled goat anti-rabbit IgG (1∶5000 dilution, Zhongshan, China). After washing, bound HRP on the membranes was detected by ECL (Pierce).

### Fluorescence confocal microscopy

SW480 cells were grown on coverslips for 12 h and then incubated with 25 µM ATRA. Subsequently, the cells were fixed in a 1∶1 methanol/acetone mixture (vol/vol) for 10 min at room temperature. After washing with PBS, the cells were blocked with 10% goat serum in PBS for 30 min and then incubated with the anti-ICAM-1 antibody (1∶100 dilution) for 2 h at room temperature. The coverslips were washed with PBS containing 0.1% TWEEN 20, incubated with fluorescein isothiocyanate (FITC)-conjugated goat anti-mouse IgG (1∶100 dilution, Biotium) for 1 h at room temperature and then counterstained for nuclei with 4′,6-diamidino-2-phenylindole (DAPI, Sigma) for 30 min. Coverslips were washed, mounted and examined under a confocal microscope (Zeiss LSM 510). The experiment was conducted in duplicate.

### Flow cytometry

For the analysis of ICAM-1 expression at the cell surface, a total of 1×10^6^ SW480 cells were treated with 25 µM ATRA for 48 h. After washing in ice-cold PBS containing 1% fetal calf serum (FCS) and 0.5% sodium azide, the cells were incubated with the anti-ICAM-1 antibody (1∶100 dilution) on ice for 30 min, followed by washing with PBS containing 1% FCS and 0.5% sodium azide. Then the cells were incubated with FITC-conjugated secondary antibody (1∶100 dilution, Biotium) for 20 min on ice. After washing, the cells were resuspended in 1% paraformaldehyde. For the analysis of total ICAM-1 expression in the cells, the cells were fixed with Intracellular Fixation Buffer (eBioscience) overnight at 4°C, treated with Permeabilization Buffer (eBioscience) and stained according to the manufacturer's instructions. The stained cells were analyzed on a FACSCalibur flow cytometer (BD Bioscience). The experiment was conducted in duplicated.

### Adhesion assay

SW480 cells were fluorescently labeled with 20 µM 1,1′-dioctadecyl-3,3,3′3′-tetramethylindocarbocyanine perchlorate (DiI, Beyotime Institute of Biotechnology) for 20 min at 37°C and subsequently washed in DMEM containing 0.1% BSA. The labeled cells (10^5^ per well) were co-cultured with HUVEC monolayer in six-well plates at 37°C for 3 h. After incubation, non-adherent cells were removed by washing with PBS and adherent cells were measured under a fluorescence microscope (Nikon Eclipse TE2000U-E).

### Trans-endothelial migration of U937 cells

Migration assays were performed in transwell plates (Corning Costar Corp.) with 8 µm pore filters. HUVEC monolayers were cultured on matrigel-coated transwell filters as described previously [53] and treated with 10 ng/ml TNFα (R & D). U937 cells treated with or without 25 µM ATRA were then added to the upper chamber. At the same time, 600 µl of serum free RPMI 1640 containing 50 ng/ml IL-8 (PEPROTECH) were added to the lower chamber. Transmigration was proceeded at 37°C for 24 h. The experiment was stopped by removing the transwell insert. Transmigrated U937 cells were labeled with Dil and counted by flowcytometry using a FACSCalibur flow cytometer (BD Bioscience).

### Statistical analysis

For all real-time RT-PCR analysis and cell counting, the results are expressed as means±standards errors (SE). The differences were determined by t test, where p<0.05 is regarded as significant.
